# Initiation of Hybrid Closed‐Loop Artificial Pancreas System Improves Glycemic Control in a Hospitalized Type 1 Diabetes: A Case Report and Review

**DOI:** 10.1155/crie/8925266

**Published:** 2026-01-20

**Authors:** Shaohua Li, Pin Lv, Haosi Lu, Yang Liu, Yang Shen, Cijing Cai, Xifeng Zeng, Hongyun Lu, Yu Zhao

**Affiliations:** ^1^ Wards of Cadres, Zhuhai Clinical Medical College of Jinan University (Zhuhai People’s Hospital, The Affiliated Hospital of Beijing Institute of Technology), Zhuhai, 519000, China; ^2^ Tangtangquan (Aibaowei Biotechnology Co., Ltd.), Shenzhen, 518102, Guangdong, China; ^3^ Department of Endocrinology and Metabolism, Zhuhai Clinical Medical College of Jinan University (Zhuhai People’s Hospital, The Affiliated Hospital of Beijing Institute of Technology), Zhuhai, 519000, China; ^4^ Department of Endocrinology, Affiliated Hospital Group of Guangdong Medical University Shenzhen Baoan Central Hospital (Baoan Central Hospital of Shenzhen), Shenzhen, 518102, Guangdong, China

**Keywords:** android artificial pancreas system, closed-loop insulin infusion system, type 1 diabetes

## Abstract

**Background:**

To observe the efficacy and safety of artificial pancreas (Android‐hybrid closed‐loop [HCL] system) in patients with type 1 diabetes mellitus (T1DM), and to compare it with insulin multi‐injection therapy.

**Case presentation:**

A patient with long course of type 1 diabetes was treated with multiple injections of insulin, Android‐HCL therapy, and sequential multiple injections of insulin. The intraday glucose fluctuation (especially after lunch, before sleep, and during sleep) of the Android‐HCL system was significantly superior to that of the sequential multi‐injection insulin therapy. The administration of artificial pancreas system (APS) could significantly reduce the occurrence of Grade 2 hypoglycemia events.

**Conclusion:**

Artificial pancreases are feasible for inpatients with type 1 diabetes and may be preferable to conventional insulin therapy.

## 1. Background

Due to the pathogenesis of absolute insulin deficiency, type 1 diabetes presents difficulties in blood glucose control, high glycated hemoglobin, low achievement rate, large fluctuations in blood sugar, and frequent hypoglycemia. In order to better simulate the normal pancreatic insulin secretion pattern, reduce blood glucose fluctuations, and lower the risk of hypoglycemia in type 1 diabetes patients, the development of an artificial pancreas system (APS) for blood glucose management has emerged. The APS, also known as the closed‐loop insulin delivery system (CLS), has been under development for half a century and can currently be divided into hybrid closed‐loop (HCL) and fully closed‐loop (FCL).

At the 2021 European Association for the Study of Diabetes (EASD) annual meeting, the American Diabetes Association (ADA) and the EASD released the first “ADA/EASD Joint Consensus on Management of Adult Type 1 Diabetes” (referred to as the “Consensus”), which details the current clinical diagnosis and treatment strategies for adult type 1 diabetes mellitus (T1DM) [1]. The consensus acknowledges the safety and effectiveness of artificial pancreas in the treatment of type 1 diabetes patients, with “intelligent control algorithms, continuous glucose monitoring (CGM), and continuous subcutaneous insulin infusion (CSII)” as the three important components. Unlike traditional insulin pumps, APS can simulate the glucose response pattern of pancreatic beta cells and automatically, progressively, and continuously adjust the insulin infusion rate up and down within the preset glucose target value (or range), providing greater advantages in diabetes management.

In this case report, we discuss the effectiveness and safety of artificial pancreas treatment by applying hybrid HCL for inpatient blood glucose management in a 17‐year history of type 1 diabetes female patient. We also compare this treatment with the conventional multiple daily injections (MDIs) insulin therapy. This is also the first reported case in China of an inpatient type 1 diabetes patient using an APS.

## 2. Case Presentation

A 28‐year‐old female with type 1 diabetes for 17 years was admitted to our hospital on April, 2022. The patient started subcutaneous insulin therapy at diagnosis without systematic monitoring of blood glucose. Seven years ago, the patient experienced “pain in both feet” and the insulin regimen was adjusted to “20 IU of Novolin R subcutaneous injection twice a day, in the morning and evening.” Later, the patient herself changed the insulin regimen to “50–60 IU of Novolin R subcutaneous injection once daily” without medical advice. Thereafter, the patient suffered from frequent episodes of hypoglycemia and only received glucose supplementary treatment. In 2019, the patient began to experience blurred vision and dizziness, and the insulin regimen was regulated to “12 IU of detemir insulin injection before bedtime and 5 IU of human insulin before meals.” At present, the patient has developed left lower limb edema without an obvious cause, accompanied by mild pain in the dorsal and plantar aspects of the left foot, and was admitted to our hospital. The current insulin regimen was 25 IU of glargine insulin in the morning and 5–8 IU of human insulin before meals to control blood glucose, whereas the patient did not regularly monitor blood glucose.

On physical examination, with a body mass index of 20.5 kg/m^2^, she had left lower limb edema, symmetric and strong dorsal artery pulses in both feet. The 10 g nylon filament test was negative without abnormalities in vibration and pain‐temperature sensation.

Laboratory tests indicated an elevated glycated hemoglobin measurement of 11.9% (reference range 4.0%−6.0%). Diabetes autoantibody presented positive islet cell antibody (2.16 COI) and glutamic acid decarboxylase antibody 168.00 IU/mL (reference range 0–1.00 IU/mL). No significant abnormalities were found in fundus photography, ABI, and somatosensory evoked potentials.

On admission, the random fingertip blood glucose was 7.8 mmol/L, blood ketones were 0.1 mmol/L, and glycated hemoglobin (HbA1c) was 8.2%. The patient received intensified insulin therapy with MDI on admission, with 25 IU of glargine insulin injection before bedtime and 5 IU of human insulin injections before meals (5–5–5 IU). The MDI treatment lasted from April 23, 2022 to April 29, 2022, for a total of 6 days. The fingertip blood glucose monitoring profile from April 23 to April 29 showed that the fasting blood glucose was between 4.5 and 13.3 mmol/L, and the postprandial blood glucose fluctuated between 5.2 and 22.6 mmol/L after 2 h.

Due to the large overall fluctuations in blood glucose, hybrid HCL therapy is planned to control blood glucose fluctuations. Therefore, after detailed communication with the patient about the advantages of HCL therapy and obtaining the patient’s consent and signed informed consent form, preparations for the HCL therapy were initiated.

Regarding the selection of CGM devices, we chose the Sibionics CGM (Shenzhen, China) system, which is easy to operate, convenient to wear, and provides more accurate blood glucose data. We provided face‐to‐face guidance at the bedside and implanted the Sibionics CGM sensor into the patient’s outer upper arm on April 28, 2022. One hour later, we obtained the patient’s blood glucose curve.

There are many mainstream pumps that support HCL therapy at present, and we used the compact and lightweight Dana‐R pump. The insulin pump used for HCL construction must have Bluetooth functionality and be integrated into the HCL algorithm system to achieve a smooth Bluetooth connection.

Before setting up HCL for patients, it is necessary to configure the initialization parameters for HCL. The specific method is to calculate the initial insulin sensitivity factor (ISF), initial carbohydrate ratio (ICR), and insulin duration of action (DIA) based on the patient’s recent daily total daily dose (TDD), and preset a 24‐h basal rate.

At this point, all the necessary preparation work has been completed. After installing the insulin pump for the patient, the parameters for HCL infusion safety limits are set on the closed‐loop mobile phone, such as (1) The upper limit of the patient’s daily maximum dose (Max allowed bolus [U]), which can effectively avoid excessive insulin infusion caused by mis‐operation; (2) The upper limit of maximum basal rate (Max U/ha temp basal) intervention; (3) The limitation of active insulin (maximum basal IOB OpenAPS can deliver [U]); (4) setting of super micro bolus; (5) adaptive parameter adjustment (Autosens), etc. This is also the powerful safety feature of HCL, which minimizes the occurrence of hypoglycemia to the greatest extent while ensuring patient safety and allowing for better blood glucose control in subsequent applications.

**Figure 1:**
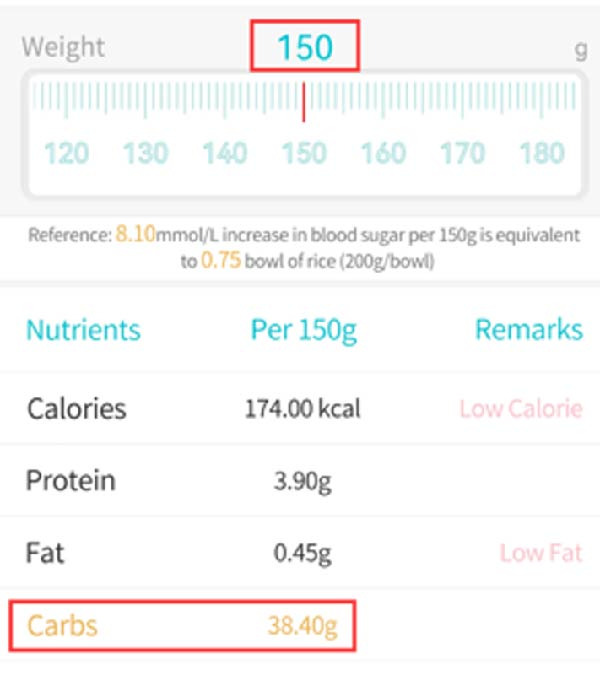
Search the food library in the Tangtangquan.

HCL algorithm construction was completed at 11:00 am on April 29, 2022. During lunchtime, the nurse weighed and calculated the carbohydrates in the patient’s main food using a food scale. The calculation process was divided into two steps: (1) weighing; (2) querying the food library of the “Sugar Circle” app to obtain the carbohydrate content of the food (Figure 1).

By manually entering the corresponding carbohydrate intake, the HCL system will automatically calculate the recommended insulin infusion dose. After confirming the infusion information, the insulin can be infused without the patient having to take out the insulin pump, thus protecting the patient’s privacy throughout the process.

After wearing the artificial pancreas for 7 days, the patient decided to temporarily switch to MDI treatment after discharge. Therefore, at 11:00 am on May 17, 2022, the patient switched to MDI therapy, with 30 units of regular insulin and 4 units of NPH insulin –4 units –4 units until discharge.

## 3. Results

Blood glucose comparison for 7 days of HCL therapy (May 11–17, 2022) and sequential MDI therapy (May 18–24, 2022) (Table 1). Compared with MDI, HCL had a higher overall TIR (*p*  < 0.05), lower TAR, TBR, and average blood glucose, but there was no statistical difference.

**Table 1:** Blood glucose comparison between HCL group and MDI group.

Group	MG (mmol/L)	TIR%	TAR%	TBR%
HCL	6.93 ± 0.99	86.94 ± 16.7	8.42 ± 12.88	4.66 ± 5.17
MDI	8.03 ± 2.17	64.89 ± 18.82 ^∗^	28.23 ± 23.87	7.41 ± 9.67

*Note:* MG, mean blood glucose; TIR, time in range percentage; TAR, time above range percentage; TBR, time below range percentage.

^∗^ *p* < 0.05.

HCL and sequential MDI AGP charts for 7 days each (Figure 2) show the following: (1) Using 6:00 am as the fasting blood glucose time point, the median fasting blood glucose levels for both treatment methods were around 7.0 mmol/L. (2) Glucose fluctuations during the day, especially from after lunch to bedtime, were significantly better during HCL treatment than during MDI treatment. This is due to the HCL algorithm’s ability to predict blood sugar increases and increase temporary basal rates. The adjustment range for this basal rate can be set according to individual patient needs, with a recommended safety upper limit of three times the daytime basal rate to ensure patient safety. Alternatively, multiple small‐dose interventions can be administered in advance through super‐micro boluses (SMB), especially when blood sugar increases significantly after meals, which can greatly reduce the time of high blood sugar exposure. (3) HCL control of glucose fluctuations during the day, especially during the entire sleep period, was also significantly better than with MDI. This is due to the algorithm’s ability to temporarily reduce basal rates when predicting that glucose trends will be below target values (which can be set according to individual patient needs, with a general population value of 5.6 mmol/L for the mean blood glucose value). In extreme cases, the temporary basal rate can even be reduced to zero to avoid hypoglycemia as much as possible. This effect is more intuitively visible in the daily glucose curves below. (4) In the “Blood Glucose Data” column, it can be seen that the HCL algorithm is superior to MDI in terms of both the estimated glycated hemoglobin (eHbA1c) value reflecting blood glucose control level and the MG, SD, and CV values reflecting glucose fluctuations. The TBR percentages (<3.9 mmol/L) for HCL and MDI were 4.8% and 5.7%, respectively. The difference in TBR (<3.0 mmol/L) was more significant, with HCL and MDI at 0.4% and 1.8%, respectively. HCL can significantly reduce the incidence of level 2 hypoglycemic events.

Figure 2HCL and sequential MDI AGP charts for 7 days each. (A) AGP profile during HCL treatment. (B) AGP profile during MDI treatment.

**Figure:**
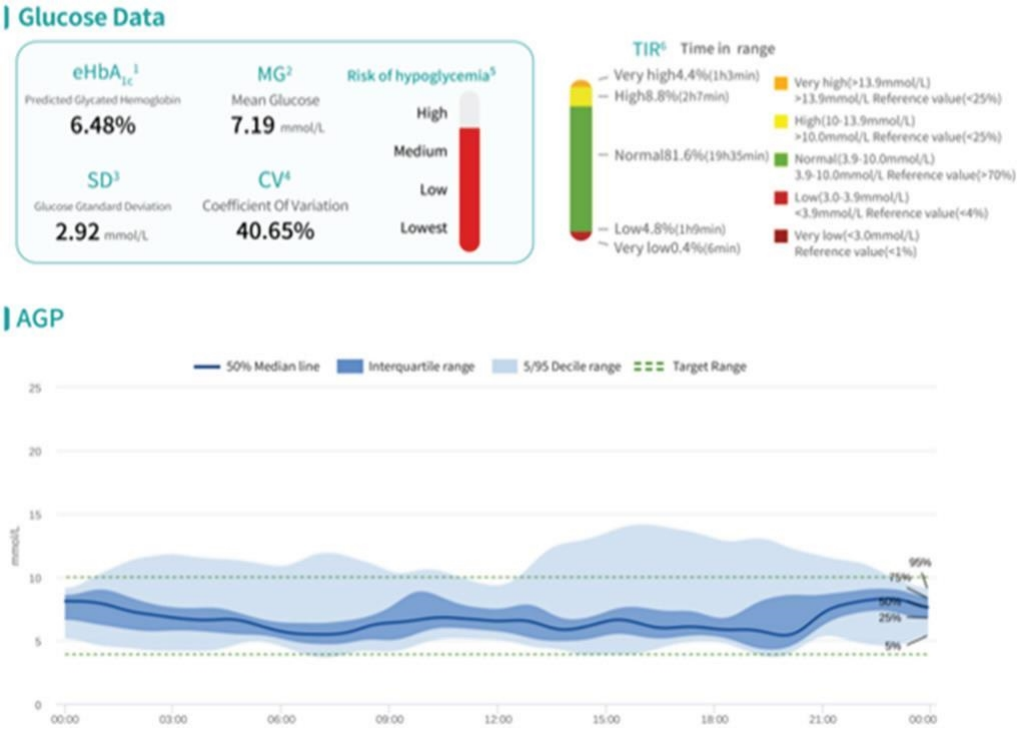
(A)

**Figure:**
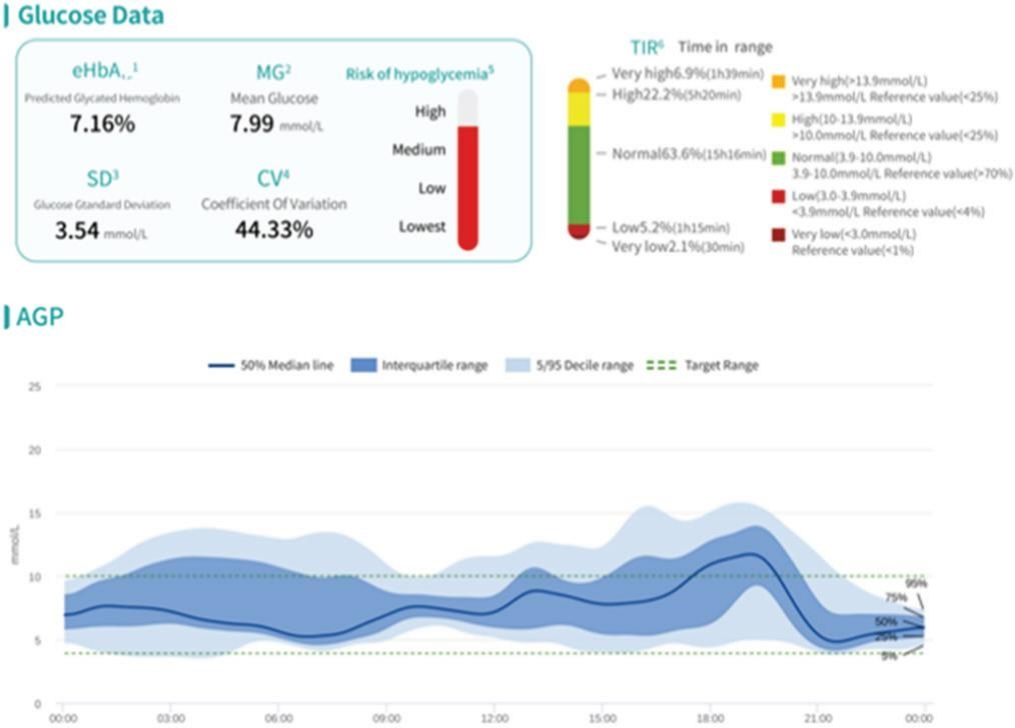
(B)

Further comparison of the daily glucose curve during HCL and MDI treatment can be seen in Figure 3: (1) There were three episodes of hypoglycemia during HCL treatment, while there were six episodes during MDI treatment (indicated by red arrows). (2) The number of high blood sugar exposures during HCL treatment was also significantly reduced (indicated by blue arrows). Although HCL intervention was implemented first and blood sugar control was relatively stable, both the doctor and the patient were aware of the blood sugar fluctuation pattern. When switched to fully manual intervention with MDI, blood sugar fluctuations increased again, and the incidence of hypoglycemia increased again. This vividly illustrates the advantages of HCL algorithms in controlling blood sugar fluctuations.

Figure 3Daily glucose curves during HCL and MDI treatment. (A) Daily glucose curve during HCL treatment. (B) Daily glucose curve during MDI treatment.

**Figure:**
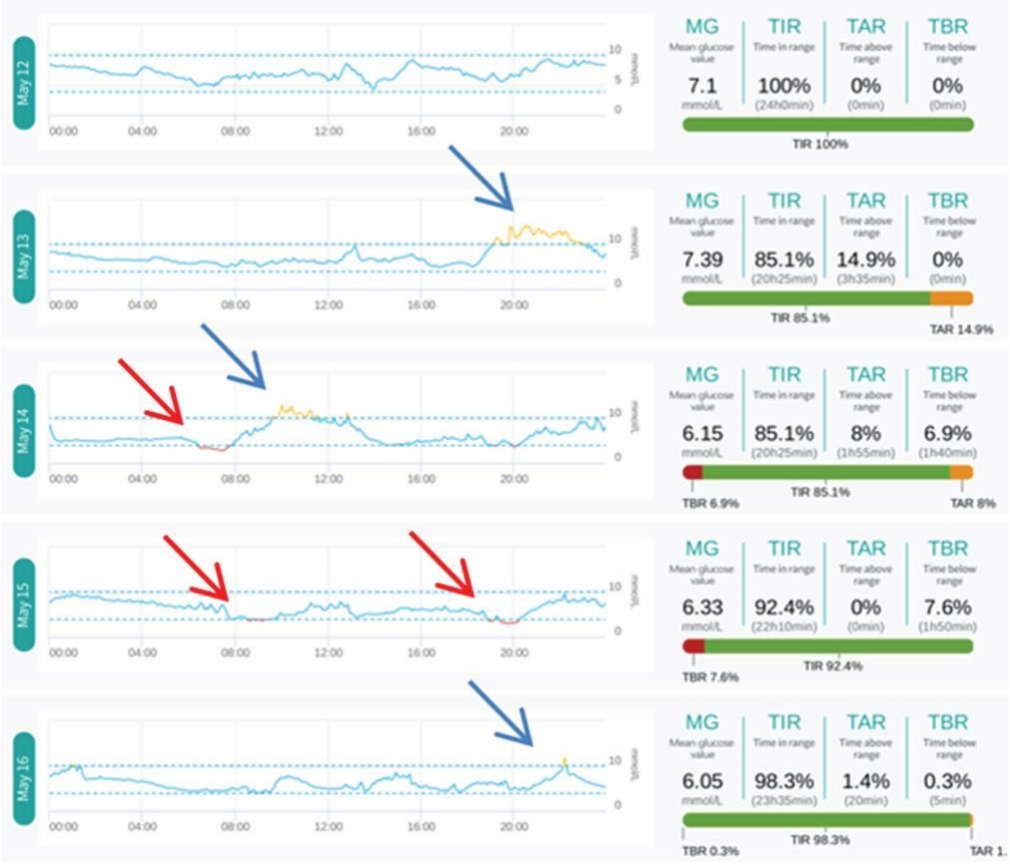
(A)

**Figure:**
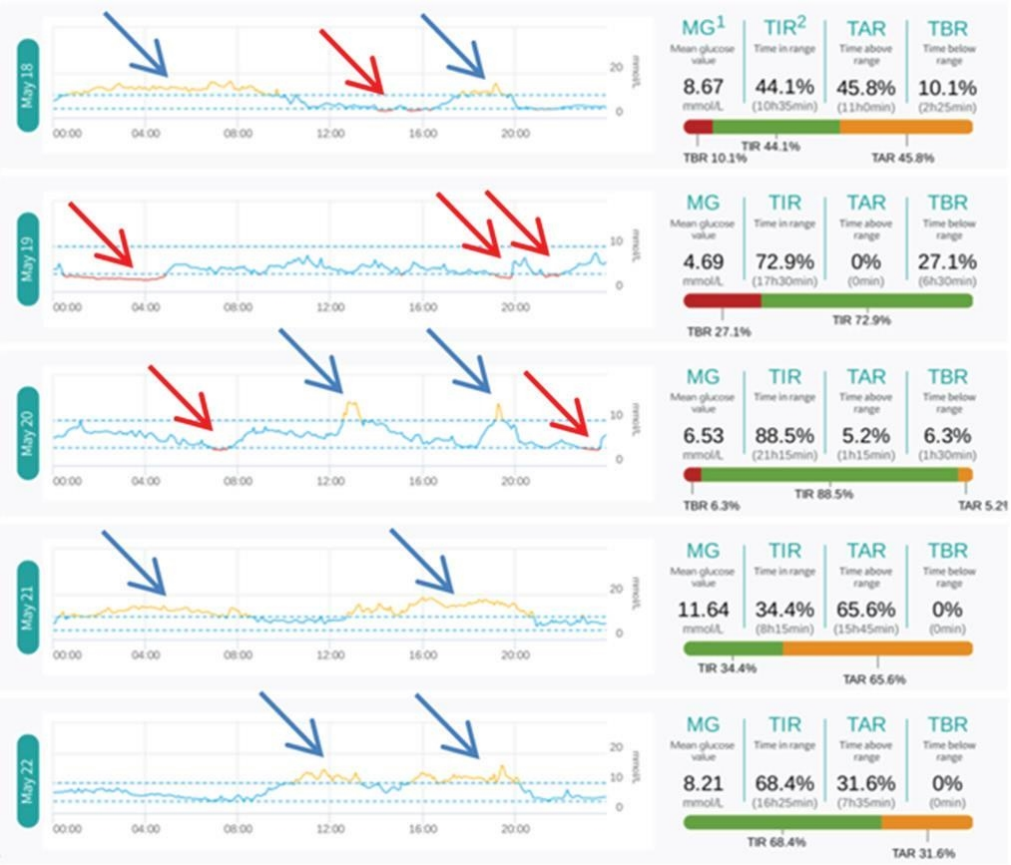
(B)

## 4. Discussion and Conclusions

Over the past decade, there have been tremendous advances in APS. The current APS landscape includes commercial products (such as Medtronic 770G/780G, CamAPS FX, Diabeloop, Control‐IQ, and Omnipod 5) and do‐it‐yourself APSs (DIY APSs), also known as open‐source AID (OS‐AID) [2]. However, none of these commercial products are currently approved or available for sale in China. As a result, individuals with type 1 diabetes, as well as hospitals and other institutions wishing to implement APS, must rely exclusively on DIY APS. These DIY APS systems, such as OpenAPS, Loop, and AndroidAPS, are affordable, allowing for personalized settings, and provide updated algorithms. Among these, AndroidAPS is the most widely used system in China.

As the first hospitalized patient with type 1 diabetes using AndroidAPS reported in China, we compared the glucose profiles during artificial pancreas treatment and sequential MDI treatment (7 days each) using AGP charts. We found that glucose fluctuations during artificial pancreas treatment, both during the day and overnight (especially from after lunch to bedtime and throughout the entire sleep period), were significantly better than during MDI treatment, and the incidence of Grade 2 hypoglycemia events was significantly reduced. In addition, the estimated HbA1c, MG, SD, and CV values were all lower with the artificial pancreas algorithm. By comparing the daily glucose curves, we also found that after sequential MDI treatment, blood sugar fluctuations increased again, including an increase in the incidence of hypoglycemia events. All of these data demonstrate the advantages of artificial pancreas algorithms in controlling blood sugar fluctuations.

OPEN APS can provide type 1 diabetes patients with higher TIR and lower HbA1c, as well as greater efficacy and safety, resulting in significant improvements in quality of life and sleep quality [2–5]. Therefore, in countries such as the United States and Europe, the value of both commercial closed‐loop systems and OPEN APS in the management of type 1 diabetes has been unanimously recognized.

The Tangtangquan team in China, which focuses on the management of type 1 diabetes, began introducing and optimizing AndroidAPS in 2018, eliminating bugs and connecting more pumps and CGMs to enable more type 1 diabetes patients with a need for this advanced closed‐loop system to use it. Currently, more than 50 type 1 diabetes patients have been set up with this system. This patient is a successful case of the collaboration between the Tangtangquan team and the endocrinology team at Baoan Central Hospital of Shenzhen in constructing an AndroidAPS system for hospitalized patients with type 1 diabetes.

In 2020, Wu et al. [6], in China, reported on the application of AndroidAPS to 15 patients with type 1 diabetes for 3 months, comparing and analyzing the results before and after application. They found that HbA1c decreased from 7.3% (SD 1.29) before application to 6.7% (SD 1.27, *p* = 0.002), TIR (3.9–10 mmol/L) increased from 75.01% (SD 10.13) to 84.28% (SD 6.92, *p*  < 0.001), and the proportion of CGM <3.9 mmol/L throughout the day decreased from 2.83% ± 1.97% 1.72% ± 0.98% (*p* = 0.011). During the use of AndroidAPS, there were no cases of severe hypoglycemia, diabetic ketoacidosis, or other serious adverse events in any of the patients. The study showed that AndroidAPS significantly improved blood glucose management compared to sensor‐augmented insulin pump therapy (SAP) [6]. However, the study population comes from outpatient clinics.

Several challenges were encountered during the implementation of DIY APS. To date, systems such as AndroidAPS remain unregulated and lack clinical validation. Consequently, future high‐quality clinical studies are imperative to facilitate the regulatory approval of APS in China. Another significant limiting factor is that insulin pumps, for both type 1 and type 2 diabetes, are currently not covered under China’s national medical insurance scheme. Given the substantial cost of these devices, this technology becomes financially prohibitive for all but a small minority of patients. Therefore, as illustrated by the case presented in this article, it is unfortunately common for patients to revert to MDI regimens following hospital discharge.

Despite that APS was regarded as “autopilot for diabetes,” autopilot does not mean that there is no need for human control. Like other technologies, there is also a learning curve for the use of an artificial pancreas. Good blood sugar still requires active management by patients, but closed‐loop systems can reduce the time and energy needed for manual blood sugar control.

**Nomenclature**
T1DM: Type 1 diabetes mellitus
APS: Artificial pancreas system
CLS: Closed‐loop insulin delivery system
HCL: Hybrid closed‐loop
FCL: Fully closed‐loop
ADA: The American Diabetes Association
EASD: European Association for the Study of Diabetes
CGM: Continuous glucose monitoring
CSII: Continuous subcutaneous insulin infusion
MDI: Multiple daily injections
ISF: Insulin sensitivity factor
ICR: Initial carbohydrate ratio
DIA: Insulin duration of action
TDD: Total daily dose
SMB: Super‐micro boluses
HbA1c: Glycated hemoglobin
SAP: Sensor‐augmented insulin pump therapy.

## Author Contributions

Yu Zhao and Pin Lv conceived and designed the study. Shaohua Li, Haosi Lu, Yang Shen, Cijing Cai, and Xifeng Zeng collected and cleaned the data. Shaohua Li and Pin Lv analyzed the data and drafted the manuscript. Yang Liu and Hongyun Lu. revised the manuscript and interpreted the results.

## Funding

This work was supported by a grant from the Guangdong Medical Science and Technology Research Foundation (Grant B2025229).

## Disclosure

All authors read and approved the final manuscript.

## Ethics Statement

All procedures were conducted in accordance with the principles outlined in the Helsinki Declaration II and were approved by the Ethics Committee of Baoan Central Hospital of Shenzhen (Approval Number: S2023‐XX‐021‐01).

## Consent

Written informed consent was obtained from the patient for publication of this case report and any accompanying images. A copy of the written consent is available for review by the editor of this journal.

## Conflicts of Interest

The authors declare no conflicts of interest.

## Data Availability

The data that support the findings of this study are available from the corresponding author upon reasonable request.
